# Undetected Visual Impairment Among Older People and Its Impact on Vision-Related Quality of Life: A Study Protocol †

**DOI:** 10.3390/nursrep15040125

**Published:** 2025-04-07

**Authors:** Carina Göransson, Jeanette Källstrand

**Affiliations:** School of Health and Welfare, Halmstad University, 301 18 Halmstad, Sweden; jeanette.kallstrand@hh.se

**Keywords:** eye assessment, healthcare centre, nursing, older people, undetected visual impairment, vision impairment

## Abstract

**Background/Objectives:** Ageing is a continuous process of physiological changes that occur over time and affect both ability and function, including vision. A major health issue for older people is visual impairment, which affects both daily activities and quality of life. Undetected visual impairment is a significant problem. Therefore, early detection is crucial to enable older people to optimise their vision and/or receive eye care. This study protocol aims to explore the prevalence of undetected visual impairment among the ageing population and its impact on their quality of life. **Methods:** This study has an exploratory design. We include participants attending a healthcare centre and participants attending an optician, aged 75 years and older, in Sweden. At baseline eye examinations, fundus photography, the National Eye Institute 25-item Visual Function Questionnaire (NEI VFQ-25) and health economic data are collected. Individual semi-structured interviews (*n* = 25–30) will then be conducted about older people’s experiences of visual impairment, both consequences and strategies for coping with visual impairment in daily life. The NEI VFQ-25 and health economic data will be collected at 6- and 12-month follow-ups. **Conclusions**: This study will provide important knowledge to facilitate early detection of visual impairment in older people, thereby providing a deeper understanding of methods to preserve visual function and quality of life despite the presence of visual impairment.

## 1. Introduction

The process of ageing is a natural, continuous process of physiological changes that occur over time, including deterioration in ocular function [1]. Such deterioration can affect the condition of an eye or its functions [2], and often develops progressively among older adults and is consequently often identified at an advanced stage [3,4]. This may be due to a lack of awareness regarding visual impairment or a tendency to underestimate symptoms [5], including presbyopia [3], diminished night vision, and challenges in adapting to varying light conditions [5]. Such impairments can have a profound impact on the quality of life in older people [3,6].

As people age, the prevalence of visual impairment increases [1,3], primarily due to uncorrected refractive errors [1,3,7,8], cataracts [1,3,7], age-related macular degeneration (AMD) [1,3,6,7,9] glaucoma, and diabetic retinopathy [1,3,7]. Moderate or severe visual impairment affects approximately 11–30% of the global population 50 years or older [1,3]. Uncorrected refractive errors have shown to rapidly increase with age, and account for over 50% of all moderate-to-severe visual impairment for people 50 years and over [8]. However, timely evaluation of uncorrected refractive errors can lead to significant improvements in vision, usually through the prescription of appropriate glasses or contact lenses [3], but it is insufficiently implemented [3,8]. However, cataracts can be treated surgically by replacing the cloudy natural lens with an artificial lens [1].

Among people aged 60 years and older, AMD is the leading cause of visual impairment and blindness and is even more prevalent in people aged 75–85 years and older [6,10]. Globally, the number of people with AMD is expected to increase from 200 million to nearly 300 million between 2020 and 2040 [10] and with regional differences [9]. In Scandinavia, the prevalence of late AMD in people aged 65 years and older is estimated to be 5.2%, and this increases with age [11]. This is further compounded among the ageing population, particularly those above 80 years of age, which is expected to result in a 75% increase in advanced AMD cases by 2040 within the Scandinavian region [11].

One study revealed that older people afflicted with AMD or cataracts exhibited a lower vision-related quality of life (VRQL) in comparison to those with glaucoma or diabetic retinopathy [12]. Advanced bilateral AMD has been found to be associated with a decline in VRQL, attributed to a reduction in contrast sensitivity [13]. Moreover, challenges associated with living with AMD have been documented, with older people reporting difficulties in adapting to daily living [14]. Furthermore, social interactions can be adversely affected, as it becomes more challenging to recognise facial expressions, even in cases of mild visual impairment [15]. Studies have indicated that visual impairments could be a risk factor for depression, other mental health issues, and dementia [3,16,17].

A study of older people receiving home healthcare revealed that approximately 21% exhibited reduced macular function in at least one eye [18]. Moreover, a review indicated that a significant proportion of older people had uncorrected refractive errors when screened for visual impairment in community settings. However, no substantial enhancement in vision was observed during subsequent follow-ups, which might be ascribed to inadequate adherence to recommended interventions among the screened population [19]. These findings underscore the ongoing need to fortify eye health services to address the unmet needs of the ageing population [3,6]. There is an urgent need to enhance accessibility and develop strategies that integrate technological resources for the early detection of visual impairment within primary care settings [3], thereby reducing preventable suffering [1] and improving outcomes for older people’s vision [19]. Consequently, there is an imperative to formulate enhanced screening assessments for the early detection of visual impairment, whether attributable to inadequate correction or age-related diseases, in older people. This is to ensure that the consequences of undetected vision loss are reduced in the ageing population. The objective is to provide earlier and more effective treatment, whilst also enhancing vision function and quality of life for older people. The identification of those afflicted with the dry form of AMD at an earlier date, with a consequent reduction in the necessity for visits to eye clinics, enables the allocation of resources to those affected by wet AMD or other age-related diseases that require specialist eye care, including glaucoma and diabetic retinopathy. Furthermore, to deepen our understanding of the impact of visual impairment on the daily lives of older people, and the strategies they employ to cope with their condition is essential in order to enhance support to those needing it. In light of the ageing population, there is a pressing need to integrate digital technological solutions into primary care and optician services, with the aim of enhancing eye health, thereby promoting enhanced vision and overall quality of life. The aim of this study is to explore the prevalence of undetected visual impairment among the ageing population and its impact on their quality of life. The sub-aims of this study are as follows:

(i) To explore the prevalence of undetected visual impairment among older people visiting a healthcare centre or an optician in the same city.
(ii) To explore visual functioning and vision-related quality of life in older people.
(iii) To explore the influence of undetected visual impairment on daily life among older people.

## 2. Materials and Methods

### 2.1. Design

The present study employs an exploratory design [20] to gain insight into undetected visual impairment in older people and also to acquire a more profound understanding of the participants’ experience of vision-related quality of life. It is a constituent element of the Interreg Europe Undetected Visual Impairment project (Uopdaget Synstab in Danish), a Scandinavian collaboration between Denmark, Norway, and Sweden, where Denmark is the lead partner. The Interreg Europe Undetected Visual Impairment project employs an exploratory design, where comparisons will be made between the sample consisting of two groups in Sweden and a corresponding sample consisting of two groups in Denmark.

### 2.2. Setting

The protocol of this study will focus on the Swedish part of the project, which will be conducted in a county with approximately 345,000 inhabitants [21], located in the southwestern region of Sweden. The Swedish part of the study includes participants who visit a healthcare centre or an optician chain in a municipality, where approximately 21% of the population is aged 65 and over [21]. In Sweden, 20.8% of the population is aged 65 and over, out of approximately 10,000,000 people [21].

### 2.3. Sample

The sample will be purposive and consecutive [20], and it will include eligible people aged 75 and over. The inclusion criteria are as follows: a minimum age of 75 years, sufficient cognitive ability to participate in the study, and the ability to understand and communicate in Swedish.

To facilitate a comparison of the two samples in the project, it is estimated by the lead partner that the Swedish part of the project will under a given time period comprise 150 participants from the healthcare centre and 50 from the optician, corresponding to the same number as the lead partner in Denmark. The sample size is based on the knowledge that over 50% of older people over 50 years [8] and 60% over 70 years [12] have visual impairment due to uncorrected refractive errors.

### 2.4. Recruitment Process

A manager of a healthcare centre in the county will be informed of the study design, and permission will be given for the study to be conducted at the same healthcare centre. The authors will inform the staff at the healthcare centre, in both verbal and written formats, of the study’s aim, methodology and process of participant recruitment. Participant recruitment will be conducted during routine visits to the healthcare centre by older people for health issues or check-ups, such as visits to district nurses, general practitioners, physiotherapists, or laboratory staff.

Additionally, recruitment will be conducted in conjunction with health discussions, which are offered to all individuals aged 75 years within the county. These health discussions are facilitated by a district nurse and an occupational therapist, and address various health issues, along with strategies for supporting older people in managing these issues. Participants will be offered the opportunity to take part during their visit; alternatively, they may suggest an alternative time for an appointment.

In addition, the head of an opticians’ chain will be informed of the study’s details before agreeing to participate. Recruitment and inquiries will involve older people visiting the optician for an initial or annual eye assessment. Consecutive samples will be recruited from both a healthcare centre and the optician chain over a given period, with two follow-ups, at 6 and 12 months. Interviews with participants will be conducted in 2025.

### 2.5. Data Collection

The present study will collect data on three occasions: firstly at the baseline, and then at 6 and 12 months’ follow-ups. The authors and the optician will conduct the data collection, and different sources of data will be used, including eye assessments, fundus photography of the central parts of the retina, questionnaires and individual interviews. In order to minimise bias, the authors and the optician will thoroughly discuss how to conduct the eye assessment and fundus examination, develop a plan, and produce written instructions.

The participants will be informed at the baseline stage about the aim of the study, that participation is voluntary, and about the right to withdraw from participation at any time without giving a reason and without an impact on their medical care. Thereafter, the participants will sign the informed consent form. Each participant will be assigned a code.

#### 2.5.1. Eye Assessment at the Healthcare Centre

The eye assessment comprises three tests: visual acuity, both distance and near, and contrast sensitivity. Visual acuity is categorised as mild, moderate, severe, or blindness, based on the visual acuity in the better eye [22]. The eye assessment will be conducted by both authors, who are both registered nurses, and one is also a specialist nurse in ophthalmic care. Participants will be required to wear their current corrective lenses for all three tests.

The standard definitions for distance visual acuity are as follows: mild visual impairment is defined as worse than ≥6/12 and equal or better than <6/18 in the better eye; moderate visual impairment is defined as worse than ≥6/18 and equal or better than <6/60 in the better eye; and severe visual impairment is defined as worse than 6/60 and equal or better than 3/60 in the better eye [22]. The assessment of distance visual acuity will be conducted using a standardised eye chart with a logarithmic scale of three metres (Hedin board in accordance with the International Standard Organization [ISO] standard), following the method outlined by Snellen [3]. This will be performed one eye at a time, with the other eye being completely covered during the test. As habitual visual acuity will be assessed, the use of a pinhole will not be employed.

Near visual acuity will be assessed using a handheld card on which paragraphs of text with a readability index of 40 are printed; this corresponds to a normal written text. The test will also be performed at the participant’s preferred reading distance of approximately 40–50 cm, while sitting [3]. One eye will be assessed at a time, while the other is completely covered during the test.

Contrast sensitivity will be assessed by means of the Mars Letter Contrast Sensitivity Test [23], which examines an individual’s ability to detect differences in luminance as it is decreased by a constant factor. One eye will be examined at a time, with the fellow eye completely occluded during the test; both eyes will then be examined binocularly. This assessment provides insight into the participant’s ‘practical’ vision, which is particularly relevant for navigating outdoor environments and noticing changes in terrain, such as stairs and curbs.

Following the completion of the eye assessment, the participants will be informed of their visual acuity, any potential need for an update to their spectacles, and general information regarding issues such as dry eyes, if applicable.

#### 2.5.2. Fundus Examination

Subsequent to the eye assessment, fundus photography of both eyes (retina) will be performed, with the macula in the centre. This will be carried out using a non-mydriatic desktop fundus camera (Essilor Retina 550); therefore, there is no necessity for the administration of eyedrops for pupil dilation. Each eye will be photographed individually. The resulting photographs will be downloaded to a laptop that is compatible with the camera and that will be used exclusively for the purposes of this study.

To ensure the confidentiality and privacy of study participants, each participant’s photographs will be pseudonymised, with the same code being used throughout the study.

#### 2.5.3. Eyecheck System

The pseudonymised (coded) fundus photographs and the results from the eye assessments, together with the participants’ age, gender and any known eye disease, will be uploaded to the Eyecheck System platform, either by authors at the healthcare centre or by the optician. Thereafter, data will be transferred via the Eyecheck System to a server that is exclusively intended for data from the Swedish part of the Undetected Visual Impairment project. The ophthalmologist will be notified by email that fundus photography images are to be assessed. By using the required individual login credentials, the ophthalmologist will gain access to the results of the eye assessments, together with the photographs of the fundus. The ophthalmologist will then assess and report any diagnoses and recommendations in the Eyecheck System, such as contacting an optician or an eye clinic. Subsequently, the authors will receive an email prompting them to log into the Eyecheck System and communicate the recommended measures to the participants

The Eyecheck System is a digital communication tool for eye healthcare that is utilised in the national health sector in Norway. The tool facilitates the secure exchange of patient information, thereby enabling diagnoses to be made without the necessity for the ophthalmologist or ophthalmic nurse to be physically present at the location in question. Access to the system is restricted to the researchers (authors) involved in this study and an ophthalmologist, who will be the only persons to receive individual login credentials from the Eyecheck System. These credentials are necessary to transfer photographs of the fundus and data regarding the eye assessment. In addition to this, the researchers also store the photographs on a server at the university, a process which also requires login credentials.

The Eyecheck System is compliant with the General Data Protection Regulation [24] and adheres to the principles of information security management ISO 27001 certification [25].

#### 2.5.4. Field Notes

It is possible that participants attending the healthcare centre may voluntarily disclose their experience of visual acuity in relation to everyday activities, which may be of importance for the study. In such cases, the participant will be requested to grant permission for the use of these data in the study, and the data will be documented as field notes.

#### 2.5.5. Eye Assessment Conducted by the Optician

Participants will be recruited at the optician’s during the initial vision assessment. Those who elect to participate in the study will undergo a comprehensive eye examination, inclusive of fundus photography, performed by a trained optician. The procedure will be comparable to that employed at the healthcare centre, encompassing visual acuity testing, fundus photography, and the administration of questionnaires. The eye assessment will employ the digital Topcon CC-100 eye chart, which utilises a logarithmic scale, at a distance of 5.5 m, similar to the chart used at the healthcare centre. However, the distance to the charts and the size of the optotype letters will be adjusted to ensure the tests are comparable, irrespective of the location.

For the assessment of near-distance acuity, a Jaeger eye chart will be employed in accordance with the procedure utilised at the healthcare centre. Contrast sensitivity will not be assessed. With regard to fundus photography, a non-mydriatic camera with the macula in the centre will be employed; this is similar to the equipment used at the healthcare centre (namely, the Topcon TRC-NW400). No eyedrops will be administered in order to dilate the pupils.

This procedure will mirror that previously described at the healthcare centre for the registration of the pseudonymised eye assessment and fundus photography in the Eyecheck System and dedicated server. The ophthalmologist will log on to the server to assess the fundus photography and eye assessment, report diagnoses and any recommended measures. The participants who will be recommended measures will be contacted and informed by the optician.

#### 2.5.6. Questionnaire

The National Eye Institute 25-item Visual Functioning Questionnaire (NEI VFQ-25) will be utilised in this study. This questionnaire explores various dimensions of perceived vision-related health in diverse groups with visual impairment, primarily including those with AMD [26,27]. It has been translated into Swedish and validated [28,29]. The questionnaire comprises 25 statements relating to visual ability or emotions in connection with various everyday activities in which vision is important [27]. Participants’ answers are based on whether they wear spectacles, contact lenses or neither in everyday life [27]. The questionnaire is divided into different categories, including health and vision from a general perspective, the presence of pain in or around the eyes, participation in various activities (e.g., social contexts, theatre, or sporting events), activities that require both near and distance vision, mental health, dependence on others, driving, colour vision, and finally, peripheral vision [27]. The response alternatives are either binary (yes/no) or ordinal for the included questions. A scale is used to rate the responses between 0 and 100, with 100 representing the best vision-related quality of life. Based on the responses, an average of the vision-related subscales is calculated, with each difference of five points being associated with a decline in visual performance [27,30].

All participants will be requested to complete the questionnaire post-eye assessment and fundus photography, digitally at the healthcare centre and on paper at the optician. The participants will be requested to complete the NEI VFQ-25 questionnaire on three occasions, during the initial assessment (baseline) and at the 6- and 12-month follow-ups. This will allow for analyses of their vision-related quality of life and health economics to be conducted.

#### 2.5.7. Health Economics

A health economics analysis will be conducted [31]. Demographic data such as the participants’ age, gender, housing, and level of education will be collected from the participants. The participants will be asked whether they have experienced any visual impairment during the last year and whether any accidents have occurred due to visual impairment. If they have, the participants will be asked whether any measures were implemented. Staff will be asked about the time required for the patient’s appointment prior to the eye assessment. Furthermore, questions will be posed to ascertain the time taken by the authors and optician to make arrangements prior to and following the eye assessment. At the 6- and 12-month follow-ups, participants will be requested to complete the NEI VFQ-25 questionnaire and will be asked whether measures such as visits to an ophthalmologist or optician were implemented, if recommended at baseline. Furthermore, the occurrence of accidents due to visual impairment will be inquired.

A cost-effectiveness analysis will be conducted, encompassing the various resources utilised [31], including the expenses associated with the initial eye assessment, such as the utilisation of resources, and the costs directly related to the activities undertaken. Other resources deemed significant in relation to the initial eye assessment and the follow-ups at 6 and 12 months will also be considered. This includes measures taken by the participants as a result of the eye assessment and the recommendations made based on the fundus photographs, such as visiting an optician or an ophthalmologist for further assessments, as well as treatment for accidents caused by vision loss.

#### 2.5.8. Interviews

At baseline, participants will be asked for permission to being contacted for an individual interview at a later date. They will also be informed that the selection of participants for the study will be based on a purposive sample, with the criteria for this sample being the socioeconomic characteristics mentioned above and the results of their eye examinations. The estimated number of interviews to be conducted is between 25 and 30.

A researcher with experience in qualitative methods and who is involved in the study for the purpose of conducting interviews will contact eligible participants. The interviews will be conducted at a location that is chosen by the participants and that is undisturbed. The interviews are primarily planned to be conducted face-to-face, but they can be conducted by telephone or digitally (i.e., via Zoom) if the participants prefer. Prior to the commencement of the interview, the participants will receive both oral and written information from the interviewer, encompassing the purpose of the study, the possibility of interrupting their participation without explanation, and the opportunity to ask questions. The interviewer will ensure that the participants are apprised of the research team’s contact information in the event that any queries arise post-interview.

The interviews will be based on a thematic interview guide with open-ended questions, where follow-up questions can also be asked depending on what emerges [32]. The following themes will be explored: their path to professional help for their eyes, experiences of visual impairment in daily life, and their strategies as well as self-image in relation to visual impairment. During the interviews, participants will be invited to describe their personal circumstances and share their experiences of what visual impairment means to them in relation to their daily activities. Each interview is expected to last between 30 and 60 min and will be recorded digitally using a Dictaphone, subsequently transferred to storage media both without and with an internet connection.

### 2.6. Ethical Considerations

The present study was approved by the Swedish Ethical Review Agency (protocol code 2023-07706-01) on 7 February 2024. The study will adhere to the ethical standards of the Declaration of Helsinki [33]. In order to protect the privacy and confidentiality of the participants’ personal information, the GDPR [24] and the Swedish Act concerning the Ethical Review of Research involving Humans [34] will be adhered to.

Prior to participation, all participants will receive oral and written information regarding the study, its aims, and the option to withdraw their participation without affecting their care at the healthcare centre or their contact with the optician. Additionally, they will receive information regarding the management of their personal data in accordance with the GDPR, the measures employed to ensure participant confidentiality, and assurances that no individual outside the research group will have access to the data. Following this information, written consent will be obtained from the participants. Prior to the commencement of the study, no established relationship will exist between the researchers or optician and participants.

All data will be coded and locked in a safe at the university, separated from the code key. The coded questionnaire will be answered digitally at the healthcare centre and on paper at the opticians at baseline and at the two follow-ups via postal mail. Researchers, opticians and ophthalmologists will receive individual and unique credentials to log into the Eyecheck System, which has a dedicated server for this project. Access to the data will be restricted to researchers affiliated with the study. The digital data will be stored at Sunet (Swedish University Computer Network) specifically for Swedish researchers. The Swedish Research Council is responsible for Sunet and ensures that it is operated according to government guidelines. Research materials are to be retained for a period of ten years following the completion of the principal investigator’s project.

### 2.7. Data Analysis

The Institute for Health Economics (IHE) in Sweden will provide support in the form of health economic analyses. The results of the NEI VFQ-25, at baseline and the 6- and 12-month follow-ups, will be included in the health economic data analysis. Both descriptive and analytical statistics will be adapted to the data level and data distribution [35]. The analyses will be carried out by researchers with experience in statistical analysis.

The data collected during the interviews will be analysed via a qualitative content analysis method with an inductive approach [36,37]. Both the authors and the researcher conducting the interviews will be involved in the analysis, and they all have experience in qualitative methods. Prior to the analysis, the interviews will be transcribed verbatim. The data will then be analysed in different steps. During this process, the meaning units, corresponding to the aim of the study, will be extracted from the transcribed text by the researcher conducting the interviews. These will be condensed into shorter sentences without distorting or losing their content. The data will be further analysed and coded to describe the content of the meaning units during discussions with the researcher conducting the interviews and one of the authors. The codes will then be compared and contrasted to identify both similarities and differences. These will subsequently be categorised based on their content to reflect the main findings of the interviews [36,37]. Should any disagreement arise during the coding or categorisation process, the second author will be included in the discussion to reach a consensus [37]. Finally, the researcher conducting the interviews and the authors will discuss the data analysis using a reflexive approach, thereby strengthening the credibility of the study [36].

## 3. Discussion

This study will explore the prevalence of undetected visual impairment among the ageing population and its impact on their quality of life. Combining quantitative and qualitative data, this study aims to provide a comprehensive understanding of the prevalence of undetected visual impairments and age-related diseases, and their impact on the daily lives of older people.

The findings will enhance our comprehension of how older people perceive their visual impairment and its effect on their daily lives, and which strategies they employ to address this health issue.

The study will gather data on three occasions, offering insights into the impact of an intervention (i.e., an eye assessment and fundus photography) on the implementation of measures (i.e., updating spectacles or consulting an optician or ophthalmologist) to enhance visual ability in older people. The health economics data will also provide insights into whether such recommendations were heeded and/or whether any accidents occurred due to visual impairment. It is well-known that falls are common in people with visual impairment [3] and AMD [38]; in addition, the treatment for AMD is costly [39]. Recent research has indicated that older people do not implement the recommended measures [19]. The present study aims to enhance understanding of older people’s vision-related quality of life and how it can be supported through the implementation of two follow-ups. The rationale for this is that comparisons over time can provide a more complete understanding [20].

The NEI VFQ-25 was deemed a relevant choice for the study, as it has been developed and validated for people with visual impairment and AMD [26,27], and it has been translated and validated in a Swedish context [28,29]. Furthermore, recent research has shown positive correlations between the Danish version of the NEI VFQ and VRQL and the loss of macular function in patients with early AMD [40].

This study has some limitations. The two settings (i.e., healthcare centre and optician) will have differing baseline assessment tools and camera types. At the healthcare centre, distance visual acuity will be assessed by a Hedin board at 3 metres; in comparison, a digital eye chart at 5.5 metres will be used at the optician. This choice is pragmatic in nature, but the tools are being tested and validated scientifically, ensuring their comparability. Despite the use of different visual acuity charts in this study, both charts utilise a logarithmic scale. The Early Treatment Diabetic Retinopathy (ETDRS) chart is not applicable in the current study, as it requires a specific distance [40] and a longer assessment duration [41]. For the assessment of near visual acuity, different tools will be employed for pragmatic reasons. At the healthcare centre, handheld cards will be utilised, and the optician will employ a digital Jaeger chart. These charts are similar, ensuring that the participants’ results will be presented in readability scores in both settings, thereby facilitating the comparison of results. The reasons for using different charts for distance and near visual acuity is both that they are used in Swedish eye care and based on a prior study in Sweden [42]. It should be noted that the optician will not be able to conduct contrast sensitivity testing; however, given its status as a crucial metric of practical vision [23], particularly in the early stages of AMD [6] and for VRQL in AMD [13], it was deemed essential to include it in the study. This decision was made for all participants at the healthcare centre.

The two fundus cameras are comparable in terms of functionality. It would have been optimal to use exactly same fundus camera. However, due to practical constraints, these two will be used. For instance, the Topcon TRC-NW400 are deemed comparable to other non-mydriatic cameras [43]. The fundus cameras to be employed in this study will facilitate the transfer of fundus photography to an ophthalmologist via the Eyecheck System. This approach is expected to facilitate earlier diagnosis of age-related diseases in older people and more expeditious treatment of wet AMD in clinic settings. This procedure is consistent with the objective of enhancing the accessibility of better eye-health services [3].

The identification, information, and recruitment of eligible participants will be conducted during visits by healthcare professionals at healthcare centres or opticians. However, it is acknowledged that healthcare professionals may face considerable workloads, which could potentially influence their selection of participants for enrolment. Furthermore, participants may opt to decline responses to the NEI VFQ-25 and health economics questionnaire, which will be distributed via postal mail at the 6- and 12-month follow-ups. This may result in attrition, leading to missing data and potential implications for the study’s generalisability [20]. Finally, it should be noted that the present article constitutes a revised and expanded version of a poster entitled “Undetected vision loss among an older population—a threat to healthy aging?”, which was presented at a conference at the 6th Nordic Conference in Nursing Research, Stockholm, Sweden, 2–4 October 2024 [44].

## 4. Conclusions

This study is expected to provide insights into the prevalence of undetected visual impairment in older people who attend healthcare centre and optician. Utilising a comprehensive screening strategy that incorporates an eye assessment employing a digital technology system in two distinct settings, the findings will elucidate the necessary measures to enhance visual acuity in this population. The study’s findings may offer valuable insights into strategies to enhance accessibility of eye-health services and promote optimal eye health and vision in older age, with the aim of maintaining quality of life and supporting healthy ageing. Utilising both quantitative and qualitative methods, this study will contribute to a comprehensive understanding of the early detection of visual impairment, thereby facilitating a deeper understanding of how to preserve visual functioning and quality of life despite the presence of visual impairment.

## Data Availability

No new data were created or analysed in this study protocol.

## Public Involvement Statement

We will have a reference group which will be invited to receive information on the study and provide comments during the study period. The reference group will consist of both healthcare professionals with experience of eye health and people older than 65 years.

## Guidelines and Standards Statement

This manuscript was drafted against the STROBE statement-checklist for observational research.

## Abbreviations

The following abbreviations are used in this manuscript:

AMD	Age-related macular degeneration
ETDRS	Early Treatment Diabetic Retinopathy
IHE	Institute for Health Economics
ISO	International Standard Organization
NEI VFQ-25	National Eye Institute 25-item Visual Function Questionnaire
VRQL	Vision-related quality of life
